# Monkeypox Virus (MPXV) Transmission Dynamics in Neighboring Countries as a Potential Threat to Kazakhstan

**DOI:** 10.3390/v18070739

**Published:** 2026-07-03

**Authors:** Lespek Kutumbetov, Balzhan Myrzakhmetova, Olga Chervyakova, Kuandyk Zhugunissov, Askhat Myngbay, Alma Temirzhanova, Arman Issimov, Gulnur Admanova, Maral Bakytzhanova, Samal Almat, Gulya Issengaliyeva, Ayauzhan Shakhmanova

**Affiliations:** 1 Research Institute for Biological Safety Problems, Gvardeiskiy 080423, Kazakhstan; lespek.kutumbetov@mail.ru (L.K.); balzhan.myrzakhmetova.78@mail.ru (B.M.); olya.chervyakova.79@bk.ru (O.C.); kzhugunissov@mail.ru (K.Z.); 2 Department of Biology, K. Zhubanov Aktobe Regional University, Aktobe 030000, Kazakhstan; g.admanova@zhubanov.edu.kz; 3 Department of Zootechnology, Genetics and Breeding, Toraighyrov University, Pavlodar 140000, Kazakhstan; 4 Department of Ecology, K. Zhubanov Aktobe Regional University, Aktobe 030000, Kazakhstan; maralbakhytzhanova@gmail.com (M.B.); samal.almat@bk.ru (S.A.); gissengaliyeva@mail.ru (G.I.); ayauzhan.shakhmanova@mail.ru (A.S.)

**Keywords:** monkeypox virus, epidemiology, transmission dynamics, emerging infectious diseases, Central Asia, Kazakhstan

## Abstract

The global outbreak of monkeypox virus (MPXV) demonstrated qualitatively new epidemiological characteristics of the infection, significantly different from previous outbreaks, mainly limited to the regions of Central and West Africa. The scale and speed of the spread of the virus necessitated a revision of existing ideas about the transmission routes of MPXV and its epidemiological potential. Human-to-human transmission of MPXV has traditionally been considered to be limited and self-extinguishing. However, the data obtained during the 2022 outbreak indicate the formation of stable transmission chains and an increase in the effectiveness of anthroponotic spread. This work is devoted to an analysis of the dynamics of transmission of MPXV with an emphasis on its potential to spread to Kazakhstan from neighboring countries and an evaluation of preparedness, eventually aiming to raise awareness in the scientific community of Central Asia.

## 1. Introduction

Previously, the monkey pox virus (MPXV) was considered a rare disease transmitted from animals to humans [1]. However, it has now become a serious health problem for people around the world. The fact that the virus began to spread in regions where it had not been before showed that it can find new transmission routes and circulate in certain groups of people [2]. It is especially difficult to fight the disease when people become infected but have no symptoms, which makes it very difficult to track and contain its spread [3]. The situation is aggravated by the fact that the global spread of MPXV occurred against the backdrop of the COVID-19 pandemic, which created an additional burden on health systems and limited the ability to respond in a timely manner. As a result, a new model of infection spread was formed, characterized by the transition from local zoonotic outbreaks to complex global networks of interpersonal transmission. From an etiological point of view, MPXV is a zoonotic orthopoxvirus that has been characterized by transmission from animals to humans [4]. Nevertheless, the accumulated data indicate its ability for long-term and sustained transmission in human populations. In particular, studies conducted in the Democratic Republic of the Congo (DRC) have confirmed the possibility of prolonged circulation of the virus within households and local communities [5]. A significant contribution to the increase in the incidence was made by the cessation of vaccination against smallpox, which provided cross-immunity to MPXV. Several decades after the completion of vaccination programs, a significant increase in the number of cases of the disease was recorded, especially among young age groups without immune protection [6]. Thus, the evolution of the epidemiological profile of MPXV is characterized by a transition from sporadic zoonotic cases to sustained interhuman transmission on a global scale. Current trends indicate the need to review existing epidemiological surveillance strategies and develop new approaches to control the spread of this infection. This review summarizes the current global situation of MPXV, global epidemiology and the preparedness of Kazakhstan.

## 2. Evolution of MPXV Epidemiology

Genetically, the MPXV is classified into two main clades, namely clade I (Congo basin) and clade II (West Africa) [7]. Clade I is associated with a higher mortality rate (up to 10%) and a greater ability to transmit from person to person, whereas clade II is characterized by a milder clinical course and less contagiousness [8]. The main route of infection in endemic regions was associated with direct contact with infected animals; however, a number of studies indicate the possible role of additional transmission factors, including contact with contaminated surfaces and, in some cases, potential aerosol transmission under certain conditions [9]. However, these mechanisms played a secondary role compared to direct contact.

The clinical picture included typical fever, lymphadenopathy, and a characteristic rash [10], while the mortality rate in most cases remained lower than during the 2022 outbreak [11]. Until 2022, the largest outbreaks were recorded in Nigeria and the Democratic Republic of the Congo. Of particular importance was the outbreak in Nigeria in 2017, which became a kind of harbinger of changes, since during this period there was a more active spread of the virus in urban environments [12]. According to epidemiological data of that time, the base reproductive number (R0) of the virus did not exceed one [13]. This indicated that without regular import of the pathogen from its natural reservoir, the virus did not have sufficient ability to maintain stable transmission in the population solely through interpersonal contacts.

Human-to-human transmission was limited to short chains involving a small number of consecutive cases. Mathematical models of that time confirmed that in the absence of constant contact with a natural reservoir, the virus is unable to maintain long-term epidemic chains in the human population. However, a retrospective analysis shows that against the background of a decrease in collective immunity after the cessation of vaccination against smallpox, the virus gradually adapted to the human population.

The unprecedented scale and speed of spread caused by the clade IIb strain allowed the virus to reach more than 123 countries from 2022 to 2024, showing an exponential increase in incidence, independent of its natural reservoir [14]. The meta-analysis revealed a significant change in the demographic picture: the main risk group was people aged 30–40 years, which is explained by their lack of cross-immunity from vaccination against smallpox [15].

**Figure 1:**
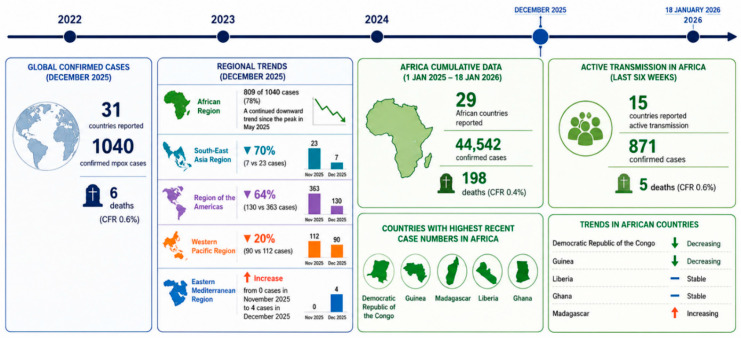
Timeline of major MPXV epidemiological events (2022–2026). Notes: Data for December 2025. Source: WHO-MPXV situation reports. Illustration was created by GPAI.

**Figure 2:**
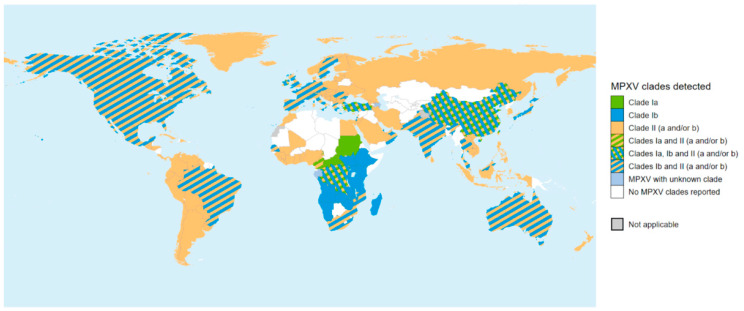
Map of the MPXV distribution worldwide according to WHO [20].

International transport hubs, mass events, and dense social media have contributed to the rapid spread of the virus, especially in Europe and Saudi Arabia [16,17]. The sharp spike in morbidity recorded by the CDC, which surpassed all historical highs, exposed new risks of globalization and required the urgent creation of international monitoring systems [18]. The change in transmission routes and geographical distribution (Figure 1 and Figure 2) has led WHO to recognize the situation as a public health emergency of international importance, emphasizing the need for a deep scientific understanding of the factors that led to such a rapid trend [19].

Earlier in the endemic regions of Africa, the infection spread among the entire population through household contact and interaction with rodents; in 2022, the vast majority of cases (about 98% of 528 in 16 countries) occurred in men who have sex with men (MSM) [20,21,22]. This observation led to an active study of the role of close physical contact during sexual intercourse as the main route of transmission of the virus. Along with the change in transmission routes, the clinical picture has also changed. Instead of the classic generalized rash, fever, and severe lymphadenopathy typical of past outbreaks, atypical and localized manifestations began to prevail. The average age of the patients increased, and the symptoms were more often manifested in the form of anogenital lesions, proctitis and pharyngitis, which could precede the general signs of infection or occur without them [10]. Isolated lesions in the genital and anus areas were often accompanied by severe pain and mucosal lesions, including ocular complications such as keratitis and conjunctivitis [23].

The diagnosis was complicated by the variability of skin manifestations: although the virus passed through the typical stages of rash development, the rashes could be limited to one area of the body [24]. The high concentration of viral DNA in the affected skin confirms the role of direct contact. Cases of asymptomatic carriage recorded in Belgium indicate the possibility of latent transmission [25]. These changes in the clinical picture and demography have complicated the initial diagnosis and highlighted the need to adapt diagnostic criteria and treatment protocols to the new realities of MPXV interaction with humans.

MPXV differs from other orthopoxviruses in its flexibility in transmission, successfully spreading through direct contact, airborne droplets and through infected objects [26]. The high prevalence of anogenital lesions and the detection of viral DNA in semen samples provided serious arguments in favor of this hypothesis [27,28]. At the same time, a high concentration of viral DNA in saliva indicates that even short-term close contact (for example, kissing) may be sufficient for infection [29]. Despite the dominance of direct skin-to-skin or skin-to-mucosa contact, the virus remains highly resistant in the external environment, which facilitates its indirect transmission through fomites, i.e., contaminated bedding, clothing, or household items [30]. Skin lesions acting as the main reservoir of the virus, especially in the early stages of infection, provide a high viral load necessary for the successful introduction of the pathogen through microtrauma of the skin or mucous membranes [31]. The development of the disease is due to the initial stage of pathogen replication at the site of initial introduction, followed by rapid spread to regional lymph nodes. This process is key for the subsequent systemic course of infection.

## 3. MPXV in Kazakhstan

It is officially confirmed that no cases of monkeypox were registered in Kazakhstan in 2022–2026 [32]. The Ministry of Health of the Republic of Kazakhstan and border control points have strengthened preventive measures aimed at preventing the import of the virus: border screening of potential patients, the introduction of algorithms such as the coronavirus procedure, training of medical personnel and analysis of trade in goods have been launched [33]. Currently, the laboratory infrastructure created within the framework of COVID-19 is being re-adapted to monkeypox [34].

In China, the first imported case of monkeypox was detected in September 2022 [35], and the first locally transmitted case was registered in May 2023 [36]. According to current data, by the end of 2024, the total number of MPXV cases in China exceeded 2000, all of which were viral lineages belonging to the IIb subclade [37]. In January 2025, WHO reported that five cases of subclade Ib had been identified in China—one of which was linked to travel to DR Congo, and the others were close contacts [38]. This suggests that China is not currently experiencing a period of epidemic instability but that there are different strains of the virus.

The first case of Mpox clade I strain in India was reported on 23 September 2024. A 38-year-old patient was found to be infected with the IMR (clade 1b) strain after returning from the United Arab Emirates [39,40]. On 22 August 2024, one case of clade Ib was confirmed in Thailand; the patient is believed to have come from Europe and contracted the virus in a jungle area [41]. In February 2025, the United Arab Emirates (UAE) also reported its first case of clade Ib [42]. Kazakhstan’s neighboring countries, such as Uzbekistan, Kyrgyzstan, Turkmenistan, Tajikistan and Mongolia, have not reported any cases; their authorities are stepping up surveillance and public awareness [43,44]. In Pakistan, a local epidemic began in March–April 2026 among newborns with weak adaptive immunity (Sindh province) [45]. At that time, 249 suspected cases were registered, 29 of which were confirmed by PCR, and eight deaths were recorded [46]. All of them belonged to the Ib clade strain [47]. Several imported cases of Ib strain were also recorded in other regions of Eastern Europe and the CIS, where frequent surveys are carried out (for example, the first two cases of Ib strain in Germany and Sweden in August 2024) [48].

## 4. Examples of Imported Cases to Neighboring Countries and Genomic Surveillance

The first case of Mpox in Russia was registered in 2022, where a 28-year-old man who had returned from Europe had symptoms of Mpox [49]. The patient presented with a widespread rash, and laboratory testing revealed viral DNA in skin lesions, nasopharyngeal swabs, and sputum. Genomic analysis showed that the virus belonged to clade IIb B.1, which is associated with the 2022 global outbreak. The identified strain had a high genetic similarity to viruses reported in the United States, Portugal, Germany, Ireland, and Peru. In addition, although several mutations were identified in the viral genome, no changes associated with resistance to the drug Tecovirimat were found.

The authors noted that Mpox infection can sometimes be transmitted by asymptomatic individuals and that the virus is likely to be shed even after clinical symptoms have resolved. The genetic similarity of the strain identified in Russia to viruses in other countries also indicated that they may have common epidemiological links [49]. Moreover, in 2026 there were three cases registered in St. Petersburg, and two cases in the Moscow region. There is a lack of information regarding the type of clade and further follow-up information, and two cases of clade IIb C.1.1 are known of from registered cases in February 2026 [50].

In 2025, the first imported case of monkeypox class Ia was recorded in Shandong Province, China. The patient was a 34-year-old man who had worked in the Democratic Republic of the Congo. He sought medical help with symptoms such as fever, rash, and enlarged lymph nodes. Based on his travel history and clinical symptoms, the patient was isolated, and MPXV infection was confirmed by laboratory tests. Genomic analysis showed that the detected virus belonged to the MPXV clade Ia. The strain showed high genetic similarity to viruses registered in the Republic of the Congo and was found to have a common ancestor with the strain identified in Ireland. These results demonstrated the persistence of international transmission chains of Mpox virus and the importance of border epidemiological surveillance [7].

This kind of case makes the spread risk of MPXV to Kazakhstan high, as Russia and China are the closest neighboring countries. Despite all the measures taken by these neighboring countries, there are still infection cases that are being registered. Currently, there are many non-endemic countries that have reported dozens of MPX cases with different mutations [34]. This raises concerns about the effectiveness of existing vaccines, including vaccines being produced in Kazakhstan. According to GISAID, neighboring countries and those most frequently visited by Kazakhstani people are showing a prevalence of clade IIb compared to other clades of MPXV (Table 1) [7].

Genomic analysis of cases in the United States identified two distinct monkeypox virus lineages: the dominant B.1 lineage associated with the 2022 global outbreak and the rare A.2 lineage [51]. Comparative mutational analysis revealed a clear predominance of GA→AA transitions, characteristic of APOBEC3 cytosine deaminase activity in host cells, in MPXV clade IIb strains circulating since 2017. This mutational pattern has not been identified in other MPXV clades. These findings suggest that APOBEC3-mediated editing has emerged as a major and recurrent driver of MPXV evolution and genetic diversification during the current outbreak [52].

Mutations in the viral DNA polymerase in the monkeypox virus genome may enhance the virus’s ability to evade the immune system. During the current outbreak, there has been a marked increase in mutations associated with the APOBEC3 enzyme [52]. This enzyme induces the formation of point mutations during viral replication and reduces the efficiency of DNA proofreading mechanisms. As a result, the genetic variability of the virus accelerates, increasing its evolutionary adaptation and spreading potential.

The genome of monkeypox virus shows high similarity to other orthopoxviruses, with the highest level of genomic identity observed with Variola virus. Phylogenetic analysis showed that sequences of the West African clade are closely related to isolates from Portugal and Belgium. While approximately 96.8% genomic identity was found between MPXV and Variola virus, 97.9% at the protein level and 97.8% at the nucleotide level were recorded between the current epidemic strains and other orthopoxviruses. The terminal genomic regions of the virus contain the majority of the genes responsible for host range and virulence, and these regions are characterized by high variability. In contrast, genes in the central part of the genome show high conservation among orthopoxviruses. In addition, the guanine–cytosine ratio in MPXV DNA is approximately 31.1% [53].

Whole-genome sequencing analyses of recent MPXV isolates have shown that current clade IIb strains differ from the 2018–2019 Nigerian isolates by approximately 40–70 nucleotide substitutions [54]. Additional genetic diversification has also been observed between different regional foci. Phylogenetic reconstructions based on maximum-likelihood and Bayesian inference methods consistently revealed a predominance of APOBEC3-related mutational signatures, particularly GA→AA and TC→TT transitions.

Most of the new SNP mutations are located in the terminal regions of the genome. These regions are rich in host-range and immunomodulatory genes. The terminal regions are more plastic than the central conserved region of the virus, while the central region encodes key replication enzymes and structural proteins.

Recurrent non-synonymous SNP mutations identified in currently circulating MPXV strains affect functionally important genes of the virus, as such mutations may alter the amino acid composition of the encoded proteins and influence their structural stability, spatial conformation, and biological activity [55]. These changes are likely to alter the function of proteins involved in viral membrane biogenesis, extracellular virion assembly, DNA replication fidelity, immunomodulatory function of cytokine decoy receptors, mechanisms of interferon response inhibition, and molecular processes that regulate the tropism of the virus for specific host cells. As a result, such SNP mutations may enhance the efficiency of virus entry into cells, its replication capacity, immune evasion, and the potential for adaptation to the human population.

Comparison of early epidemic isolates and new strains from 2024 to 2025 showed gradual diversification in regions homologous to the vaccinia virus *B21R*, *D14L*, *OPG105*, *OPG153*, and *OPG210* genes [56]. Mutations at these loci may contribute to changes in virulence phenotypes and increased adaptation to spread in the human population. The phylogenetic map shows the formation of geographically linked subclusters within clade IIb. This is consistent with the founder effect and local adaptive evolution. Although isolates from Brazil, Mexico, India, Thailand, and Colombia show distinct patterns of SNP accumulation, they retain their phylogenetic relationship to the common epidemic lineage.

APOBEC3-dependent mutations are not limited to surface antigens but have also been identified in genes that play an immunomodulatory role. In particular, changes have been observed in genes encoding TNF receptor homologs, ankyrin repeat proteins, and chemokine-binding proteins [57]. These proteins play an important role in MPXV’s immune evasion strategies. Therefore, these mutations may affect the virus’s ability to suppress the inflammatory response, distort cytokine signaling, and evade T-cell immunity.

Recent studies have also identified novel mutations located in the MPXV F8L gene. This gene encodes the viral DNA polymerase and ensures the fidelity of genome replication [58]. During studies conducted in 2024, the amino acid substitutions R25Q, E45K, A102T, F108L, and R366K were described [58]. These mutations were not found in previous MPXV strains. Structural analysis revealed that these changes are located in the exonuclease proofreading regions and DNA-binding domains of the polymerase. In this regard, the researchers concluded that these mutations may reduce replication fidelity and further enhance the mutation rate of the virus.

Structural changes in the polymerase are likely to contribute to the increased genetic plasticity of MPXV. Reduced replication fidelity increases the frequency of new mutations and allows the virus to quickly adapt to different environmental conditions [59]. This situation makes it difficult to predict the future direction of MPXV evolution. In addition, F8L mutations may also affect susceptibility to antiviral drugs. Although classical mutations associated with cidofovir resistance have not been identified at present, the possibility that new polymerase variants may contribute to the effectiveness of nucleoside analogs cannot be ruled out.

## 5. Future Directions

The emergence of a virulent clade Ib variant of monkeypox (mpox), characterized by increased frequency of household and heterosexual transmission, has once again clearly demonstrated the weaknesses of public health systems that were previously identified during the clade IIb pandemic. This situation indicates that the nature of the spread of the infection is changing, and the disease has increased the possibility of spreading among wider population groups than before.

Kazakhstan’s strategic geographical location as an important transit and transport-logistics hub connecting Europe and Asia increases the risk of rapid introduction of new virus variants into the country. In this regard, the introduction of flexible diagnostic algorithms, modern laboratory methods and genomic epidemiological monitoring systems in the healthcare system, which will allow for a rapid response to new epidemiological threats, is of particular importance. Such measures will allow for the early detection of new virus strains and the prevention of their further spread. In addition, the changing dynamics of transmission at the global level and the resurgence of clade I infections indicate the need to strengthen national laboratory capacity. Detection of imported cases before local transmission chains are established is one of the main tasks of epidemiological surveillance. This requires expanding diagnostic capabilities, improving the skills of specialists, and effective integration with international information exchange systems.

The dominance of clade Ib in different countries indicates its high level of transmissibility. This situation significantly increases the risk of the virus spreading between countries, given the intensity of international travel and the expansion of globalized transport networks. Therefore, improving epidemiological surveillance in Kazakhstan, expanding genome sequencing programs, and strengthening border sanitary control measures are important elements of ensuring public health security. In this regard, Kazakhstan should consider as a priority the introduction of rapid molecular diagnostic methods recommended by the World Health Organization (WHO) into the national healthcare system and the development of effective genomic surveillance networks that will allow for the timely detection of new mutations that form during the evolution of the virus under the influence of the APOBEC3 enzyme system. Such an approach will allow for the early detection of the emergence of new viral variants, assessment of their spread characteristics, and timely planning of anti-epidemic measures. To effectively respond to these challenges, public policymakers should pay special attention to strengthening the integration of regional health security systems in accordance with the “Strategic Framework for Strengthening the Prevention and Control of Mpox Disease for 2024–2027” developed by WHO. One of the main tasks in this direction is to increase the level of regional preparedness by developing cross-border diagnostic cooperation, accelerating the exchange of epidemiological information, and coordinating laboratory resources.

The importance of such measures is explained by the rapid spread of subclade Ib in a short period of time. Its rapid spread often exceeds the availability of specific diagnostic tests, making it difficult to identify and isolate outbreaks in non-endemic countries in a timely manner. Therefore, in addition to improving laboratory infrastructure, it is also important to strengthen international scientific and diagnostic cooperation. At the same time, the transition from a zoonotic transmission mechanism to a stable human-to-human transmission model requires improving the clinical training system in Kazakhstan. Training programs for health workers should focus on early recognition of atypical clinical signs characteristic of new, highly virulent strains of the virus. Such programs should include a variety of clinical manifestations of the disease, modern methods of diagnosis and differential diagnosis, as well as infection control principles.

In addition, training materials should pay special attention to the high pathogenicity and potential for severe complications of the clade Ib variant. Imported cases registered in Asia and Europe have demonstrated the high potential for international spread of this variant. Therefore, increasing clinical vigilance, improving epidemiological surveillance, and expanding laboratory diagnostic capabilities in Kazakhstan are important components of preparedness for new epidemiological threats.

## 6. Conclusions

Kazakhstan occupies a strategically important geographic position between Eastern Europe, the Russian Federation, China, and Central Asia, making the country highly relevant in the context of emerging MPXV transmission dynamics and viral evolution. Although Kazakhstan has not experienced large-scale endemic MPXV transmission comparable to regions in Central and West Africa, increasing international mobility, trade corridors, migration routes, and regional connectivity substantially elevate the risk of importation of genetically diverse MPXV strains.

The phylogenetic diversification illustrated in the global MPXV map is particularly important for Kazakhstan because international travel-associated transmission has become one of the primary drivers of clade IIb dissemination. Air travel networks linking Kazakhstan with Europe, the Middle East, Türkiye, Southeast Asia, and the Russian Federation may facilitate introduction of emerging variants carrying novel SNP profiles and adaptive mutations. From a molecular epidemiology perspective, Kazakhstan currently possesses limited publicly available MPXV genomic surveillance data compared with North America and Western Europe. Consequently, imported cases could remain genomically under-characterized unless systematic whole-genome sequencing programs are expanded. Implementation of genomic surveillance infrastructure within Kazakhstan would provide several important benefits. First, sequencing of imported MPXV isolates would allow early identification of lineage-specific SNP signatures associated with clade IIb diversification [60]. Second, genomic monitoring could detect mutations affecting antiviral susceptibility genes, particularly within F13L orthologs targeted by Tecovirimat [61]. Third, surveillance would enable assessment of whether locally detected strains possess mutations within membrane glycoproteins or immunomodulatory proteins associated with enhanced transmission efficiency.

**Figure 3:**
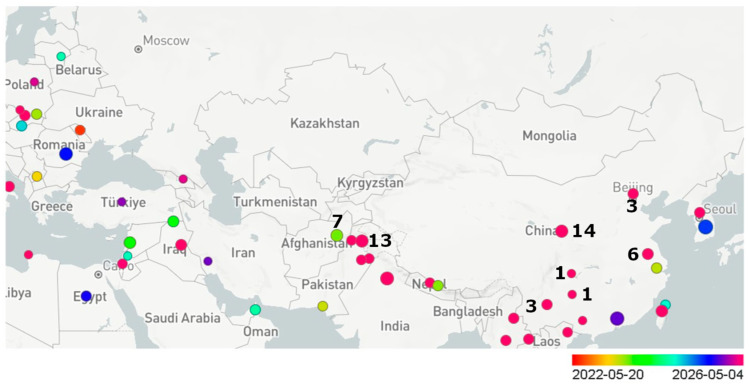
Map of newly identified variants of clade IIb in countries close to Kazakhstan. Circles represent new registered variants of clade IIb. Circle size proportional to number of variant genomes. Recency is represented by the gradient color scale below. https://gisaid.org/mpox-variants/ (accessed on 1 June 2026). The findings of this study are based on metadata associated with 9 sequences available on GISAID, via ID: EPI_SET_260701sg, DOI: https://doi.org/10.55876/gis8.260701sg (accessed on 1 June 2026).

Kazakhstan’s geographic proximity to regions with intense international movement additionally raises concerns regarding silent introduction of strains exhibiting APOBEC3-driven diversification. Because many contemporary MPXV mutations accumulate gradually during sustained transmission, even limited chains of undetected spread may facilitate establishment of regionally adapted sublineages (Figure 3).

An additional consideration involves differential vaccine preparedness across Central Asia. Existing vaccinia-derived vaccines remain expected to provide cross-protective immunity against circulating MPXV strains detected globally. However, vaccination infrastructure, diagnostic capability, and antiviral stockpiling capacity vary considerably across the region.

Although there is currently no evidence suggesting endemic adaptation of MPXV within Kazakhstan, continued global diversification of clade IIb strains emphasizes the importance of proactive surveillance rather than reactive outbreak response.

In a nutshell, there is currently no registered antiviral drug against MPXV, and no vaccinations of local people are taking place; therefore, it is very important to comprehensively monitor the new registered variants of MPXV worldwide and take precautions accordingly.

## Figures and Tables

**Table 1:** Most recent registered cases of clade IIb in countries with high interstate relations and most visited countries by Kazakhstani tourists.

		Most Recent Submission per Country	
Country	Total #Clade IIb Registered	Virus Name	Submitted
Vietnam	126	hMpxV/Vietnam/PIHCM-077/2024	4 April 2025
India	73	hMpxV/India/KL-MCL-H-184-47/2025	20 January 2026
China	62	hMpxV/China/ZJ-QZCDC-XJJ/2023	11 May 2026
Thailand	44	hMpxV/Thailand/NIC-084/2023	15 October 2024
Pakistan	12	hMpxV/Pakistan/DUHS-01/2025	27 May 2025
Russia	7	hMpxV/Russia/SPE-RII-MH277965S/2026	6 March 2026
Afghanistan	7	MpxV/env/Afghanistan/YPCH-171/2024	28 March 2025
Egypt	3	hMpxV/Egypt/MOH-NRC-0023/2022	11 February 2024
Lithuania	1	hMpxV/Lithuania/NPHSL-MB23_0015/2024	28 January 2025

Data source: https://gisaid.org/mpox-variants/7 (accessed on 1 June 2026). Multiple lineages of MPXV caused by APOBEC dependent mutations. The findings of this study are based on metadata associated with 9 sequences available on GISAID, via ID: EPI_SET_260701sg, DOI: https://doi.org/10.55876/gis8.260701sg (accessed on 1 June 2026).

## Data Availability

No new data were created or analyzed in this study.
